# Detection of Severe Acute Respiratory Syndrome coronavirus 2 (SARS-CoV-2) on frequently touched surfaces in selected areas in Accra, Ghana

**DOI:** 10.4314/gmj.v59i2.5

**Published:** 2025-06

**Authors:** Ivy A Asante, Vanessa Magnusen, Isaac Darban, Michael Oppong-Atuahene, Joseph A Quarcoo, Nana A A Ntim, Isabella Asamoah, Kwamena WC Sagoe, Joseph O Commey, Mildred A Adusei-Poku

**Affiliations:** 1 Virology Department, Noguchi Memorial Institute for Medical Research, College of Health Sciences, University of Ghana, Accra, Ghana; 2 West African Centre for Cell Biology of Infectious Diseases (WACCBIP); 3 Department of Medical Microbiology, University of Ghana Medical School, Korle-Bu, College of Health Sciences, University of Ghana, Accra, Ghana; 4 Ghana Infectious Diseases Centre (GIDC), Ghana Health Service, Ministry of Health, Accra, Ghana

**Keywords:** SARS-CoV-2, COVID-19, touched surfaces, treatment centre, Hygienic practices

## Abstract

**Objectives:**

This study aimed to detect the presence of SARS-CoV-2 genetic material on frequently touched surfaces in Accra and assess its potential infectivity. It evaluated whether detected viruses were viable, providing insights into the possible role of environmental surfaces in COVID-19 transmission.

**Design:**

A cross-sectional study with a convenient sampling approach.

**Setting:**

Conducted in clinical (two COVID-19 isolation centres and a testing laboratory) and non-clinical (two schools and two banks) settings in Accra, Ghana, from May to September 2022.

**Intervention:**

Frequently touched surfaces were sampled at two points: morning (before disinfection) and afternoon (after work). Sterile oropharyngeal swabs moistened in Universal Transport Medium were used to swab surfaces like door handles, tables, handrails, taps, benches, washrooms, classrooms, and banking halls. RT-qPCR was used to detect viral RNA, and Vero E6 cells were used to attempt virus isolation from positive samples.

**Results:**

SARS-CoV-2 RNA was detected on 6.29% (37/588) of surfaces. Morning samples showed a positive rate of 4.08% (12/294), while afternoon samples showed a rate of 8.50% (25/294). Clinical settings had higher detection rates (7.5%) than non-clinical (3.41%), though not statistically significant (p = 0.060). The testing lab showed a significant difference between morning (2.08%) and afternoon (8.05%) detections. Positive samples were most commonly found on plastics (14/37) and metals (14/37).

**Conclusion:**

SARS-CoV-2 RNA was identified on frequently touched surfaces in selected areas of Accra, Ghana. This highlights the need for thorough hygiene and disinfection practices to prevent the spread of potential viruses.

**Funding:**

Study was funded by the Noguchi Memorial Institute for Medical Research (NMIMR) through the NMIMR Office for Research Support Fund (Fund ID EC/P25421/03).

## Introduction

SARS-CoV-2, a member of the beta coronavirus family, is one of the three highly pathogenic viruses that have sparked global concern due to their zoonotic origins and ability to cause severe respiratory diseases in humans.^1^ This novel coronavirus first emerged in late 2019 in Wuhan, China, and rapidly spread worldwide, leading to the just ended COVID-19 pandemic. The Virus has proven to be highly contagious and fatal, infecting 756,291,327 people and causing 6,841,640 deaths globally.^2^

Of the over 9 million cases reported in Africa, 171,152 cases and 1,462 fatalities were reported from Ghana.^2^,^3^ According to the World Health Organisation, the virus spreads primarily between people in close contact through infected secretions such as saliva and respiratory secretions or droplets expelled through coughing, sneezing, and talking.^4^ While direct and airborne transmission of SARS-CoV-2 are commonly acknowledged as the primary modes of transmission, the significance of indirect transmission should not be underestimated, as has been documented in multiple studies. Indirect transmission occurs when respiratory droplets from infected individuals are deposited onto surfaces and objects, creating fomites. These fomites can potentially harbour viable SARS-CoV-2 virus particles for varying durations, which can range from hours to days, depending on environmental factors such as temperature, humidity, and the type of surface involved.^5^-^7^

Several studies have investigated the presence of SARS-CoV-2 RNA in air and environmental surfaces, with a focus on healthcare settings.^5^,^8^,^9^ Nevertheless, a comprehensive review study revealed the presence of SARS-CoV-2 contamination on various surfaces across different types of facilities. The study found that approximately 17.7% of samples collected from hospital settings and 10.1% of samples from non-hospital settings tested positive for SARS-CoV-2 RNA. This indicates that surface contamination with the virus is prevalent in both healthcare and non-healthcare environments, emphasizing the need for thorough hygiene practices and disinfection protocols to prevent the potential spread of the virus.^10^ Similar investigations carried out in both non-hospital and hospital settings have discovered significant levels of surface contamination, primarily on surfaces made of plastic, glass, paper, metal and stainless steel.^10^,^11^

This suggests that frequently touched surfaces in hospitals and public places could be significant sources of transmission for SARS-CoV-2. Ghana's capital, Accra, has been referred to as the COVID-19 epicentre and has the country's highest rate of SARS-CoV-2 infections.^3^ With a population of roughly 5.4 million, the area is both the most populous and the smallest in Ghana.^12^ The area is relatively dry due to its location within the dry coastal equatorial climate zone, which has temperatures ranging from 20°C to 30°C. Knowledge regarding the contamination of ambient surfaces in Ghanaian public spaces with high human traffic and concentration, such as banks and schools, with SARS-CoV-2 is limited. It is therefore crucial to assess the level of SARS-CoV-2 contamination in public spaces and healthcare settings. This study identified the presence and infectivity of SARS-CoV-2 on regularly handled surfaces at specific locations in Accra, Ghana.

## Methods

### Study design and study site

This study employed a cross-sectional design and convenient sampling to assess frequently touched surfaces in clinical and non-clinical settings across Accra, Ghana. Clinical sites included two (2) treatment centers (TC1 and TC2), and a research centre (RC), chosen for their critical roles in COVID-19 care and testing. Non-clinical settings on the University of Ghana campus encompassed high-traffic public areas such as schools and banks. These locations were selected due to their dense populations, providing insight into potential SARS-CoV-2 contamination in varied settings.

### Observations at study sites

A preliminary observational study guided the selection of sampling locations at both clinical (TC1, TC2 and RC) and non-clinical (banks and schools) sites. It involved monitoring these sites daily for one week, recording frequencies of surface touches and individual contacts. Data on hand-wash stations and cleaning practices were also collected.

### Sample Collection

Sampling was conducted from May to September 2022, coinciding with Ghana's fourth wave of COVID-19 cases. Surface samples were taken in the morning before disinfection and again approximately 7 hours after work in the afternoon. In clinical settings, swabs were collected from areas including Intensive Care Units, Isolation Wards, Kitchenettes, Nurse's Stations, Outpatient Departments, Pharmacies, and laboratory spaces. Commonly touched surfaces such as door handles, beds, floors, tables, handrails, tap handles, trolleys, drawer handles, benches, chairs, computer peripherals, medical equipment, and fridge handles were sampled. Non-clinical settings included schools and banks, with samples taken from administrative offices, teaching laboratories, washrooms, classrooms, and banking halls. Surface materials were noted, and temperature and humidity levels were monitored using digital devices placed at each sampling site for 30 minutes before recording. Oropharyngeal swabs pre-moistened in universal transport medium were used and transported at 4°C to NMIMR for molecular analysis. A total of 588 swab samples were collected (294 in the morning and 294 in the afternoon), accompanied by temperature and humidity data from each location.

### RNA Extraction and RT-PCR for detection of SARS-CoV-2

Viral nucleic acid was isolated from swabs using the QI-Amp Viral RNA extraction kit (Qiagen GmBh, Germany) following the manufacturer's protocol. Real-Time Reverse Transcriptase PCR (RT-qPCR) assay targeting SARS-CoV-2 ORF3a and N gene was performed using Veri-Q nCoV-OM (MiCo BioMed Co., Ltd, Republic of Korea) commercially available detection kit. RT-qPCR results were interpreted based on the kit protocol.

### SARS-CoV-2 Viability by Cell Culture

Virus isolation was attempted to determine the infectivity potential of samples that tested positive for SARS-CoV-2 by RT-PCR. Vero E6 cells were seeded in 24-well plates in DMEM growth media (component) at 37°C in a 5% CO_2_ incubator. After 24 hours, cells reached 80% confluency.

The culture medium was removed, and cells were washed with phosphate-buffered saline (PBS). 200µl of the positive samples were inoculated onto the cells and incubated for 1 hour. Fresh DMEM media (without FBS or antibiotics) was added to each well, and the infected cells were incubated for 3 days. Cells were examined daily for cytopathic effect (CPE). After the incubation period, the supernatant was harvested and used for molecular analysis. Samples were considered negative after three passages under these conditions: absence of CPE and negative RT-qPCR results from the third passage supernatant.

### Data Analysis

Data was analysed using GraphPad Prism software version 16.10 and Stata software version 14.

**Ethical approval:** Approval was obtained from the Scientific and Technical Committee of the Noguchi Memorial Institute for Medical Research: STC Paper 6(4) 2021-22 (25^th^ May 2022)

## Results

### Observational study

**Figure 1:**
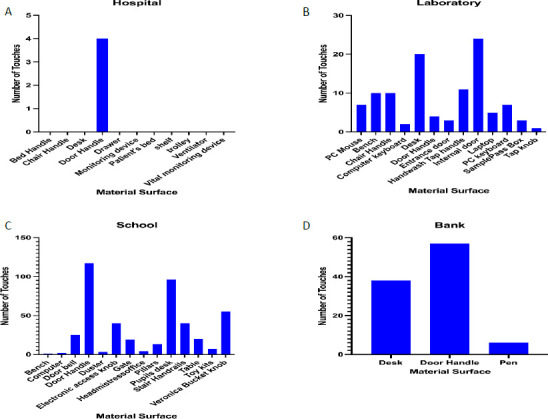
Observational study results showing frequency of touches per surface. A shows frequently touched surfaces in treatment centres, B. Research laboratory C. selected school D. Selected bank. Results show that door handles are the most frequently touched surfaces across both clinical and non-clinical setting

We visited clinical sites (treatment centres and research laboratories) and non-clinical (schools and banks) sites prior to starting the study to observe which surfaces were frequently touched. This informed the decisions of where to swab when the study began. We observed that, across all the sites, door handles were the most frequently touched surfaces. In clinical settings, door handles were touched a total of 28 times over a four-hour period, whereas in non-clinical settings, door handles were touched a total of 161 times within the same four-hour period. Again, desks were the second-highest frequently touched surfaces across both clinical and non-clinical settings (19 and 147, respectively) within 4 hours. Other areas commonly touched in clinical settings included the hand wash station tap handle (12), bench (10), chair handle (10), and the mouse of the computer (7). For non-clinical settings, other frequently touched areas included the knob on the handwash station (60), handrails for stairs (40), and the knob for electronic door access (40) (Figure 1).

### Distribution of surface samples according to study sites and areas swabbed

A total of 588 surface samples were collected from both clinical and non-clinical study sites at different time points, with 294 samples collected in the morning and 294 samples in the afternoon. At the clinical and non-clinical sites, a total of 412 samples (70.07%) and 176 samples (29.93%), respectively, were obtained. Within these categories, 364 (61.90%) samples came from the treatment centers, 48 (8.16%) from the research centre, 112 (19.05%) from our selected schools and 64 (10.88%) from banks. Samples with cycle threshold (Ct) values below 40 (<40) were considered positive, by the manufacturer's guidelines.

Overall, the prevalence of SARS-CoV-2 RNA detected on frequently touched surfaces was 6.29% (37/588). We detected SARS-CoV-2 from 4.08% (12/294) of surfaces swabbed in the morning, and 8.05% (25/294) in the afternoon (Table 1). The morning and afternoon positivity varied significantly, with the highest positivity in the afternoon (p-value = 0.027). Positive swabs were realised from 12 surfaces in the morning (10 (2.43%) from clinical and 2 (1.14%) from non-clinical settings). In contrast, 25 SARS-CoV-2 positive swabs were realised in the afternoon (21 (5.10%) from clinical settings and 4 (2.27%) from non-clinical settings). For clinical settings, SARS-CoV-2 was detected in 3.40% (10/294) of surfaces swabbed in the morning and 7.14% (21/294) of surfaces analyzed in the afternoon (p-value = 0.001). For the non-clinical sites, we detected SARS-CoV-2 for 0.68% (2/294) of surfaces swabbed in the morning and 1.36% (4/294) of surfaces analysed in the afternoon. These observations, however, were not significant (p-value > 0.001).

**Table 1:** Overall positivity of SARS-CoV-2 on Surface Samples

Characteristics	Number of Samples processed	Sites	Morning (N=294)		P-Value	Afternoon (N=294)		P-Value
			n	(% n/N), CI		n	(% n/N), CI	
**Total**	588		**12**	4.08%		**25**	8.50%	
**Categorical Site**								
**Clinical**	412		10	2.43% (1.17 - 4.42)	0.306	21	5.10% (3.18 - 7.68)	0.112
**Non-Clinical**	176		2	1.14% (0.13 - 4.04)		4	2.27% (0.62 - 5.72)	
**Study Site**								
**Clinical**								
**Treatment Center**	314	Treatment Center 1 (TC1)	9	2.87% (1.31 - 5.37)	0.868	12	3.82% (1.99 - 6.58)	0.000
**Research Lab**	48	RC	1	2.08% (0.05 - 11.06)		9	18.75% (8.94 - 32.62)	
**Treatment Center**	50	Treatment Center 2 (TC2)	0	0.00% (0.00)		0	0.00% (0.00)	
**Non-Clinical**								
**Schools**	48	School 1	0	0.00% (0.00)	0.280	1	2.08% (0.05 - 11.06)	0.629
	64	School 2	2	3.13% (0.38 - 10.83)		2	3.13% (0.38 - 10.83)	
**Bank**	34	Bank 1	0	0.00% (0.00)		0	0.00% (0.00)	
	30	Bank 2	0	0.00% (0.00)		1	3.33% (0.08 - 17.21)	
**Sampling Location**								
**Clinical**								
**Administration**	18	TC1	1	5.56% (0.14 - 27.29)	0.500	0	0.00% (0.00)	0.000
**ICU**	40		0	0.00% (0.00)		0	0.00% (0.00)	
**Isolation Ward**	102		7	6.86% (2.80 - 13.62)		5	4.90% (1.61 - 11.06)	
**Kitchenette**	16		0	0.00% (0.00)		0	0.00% (0.00)	
**Laboratory**	58		0	0.00% (0.00)		0	0.00% (0.00)	
**Nurses Station**	12		0	0.00% (0.00)		0	0.00% (0.00)	
**Outpatient Department**	40		1	2,50% (0.06 - 13.15)		0	0.00% (0.00)	
**Pharmacy**	8		0	0.00% (0.00)		0	0.00% (0.00)	
**Washroom**	20		0	0.00% (0.00)		0	0.00% (0.00)	
**Laboratory**	48	Research Center (RC)	1	2.08% (0.05 - 11.06)		9	18.75% (8.94 - 32.62)	
**Isolation Ward**	50	TC2	0	0.00% (0.00)		0	0.00% (0.00)	
**Non-Clinical**								
**Banking Hall**	26	Bank 1	0	0.00% (0.00)	0.661	0	0.00% (0.00)	0.796
**Washroom**	8		0	0.00% (0.00)		0	0.00% (0.00)	
**Administration**	12	School 1	0	0.00% (0.00)		0	0.00% (0.00)	
**Classroom**	3		0	0.00% (0.00)		0	0.00% (0.00)	
**Kitchenette**	2		0	0.00% (0.00)		0	0.00% (0.00)	
**Teaching Lab**	27		0	0.00% (0.00)		0	0.00% (0.00)	
**Washroom**	4		0	0.00% (0.00)		1	25.00% (0.63 - 80.58)	
**Banking Hall**	26	Bank 2	0	0.00% (0.00)		1	3.85% (0.09 - 19.63)	
**Washroom**	4		0	0.00% (0.00)		0	0.00% (0.00)	
**Classroom**	64	School 2	2	3.13% (0.38 - 10.83)		2	3.13% (0.38 - 10.83)	
**Sampling Area**								
**Clinical**								
**Equipment**	218	TC1	7	3.21% (1.30 - 6.50)	0.971	9	4.13% (1.90 - 7.69)	0.012
**Handle**	94		2	2.13% (0.25 - 7.69)		2	2.13% (0.25 - 7.69)	
**Surface**	2		0	0.00% (0.00)		1	50.00% (1.25 - 98.74)	
**Equipment**	34	RC	0	0.00% (0.00)		5	14.71% (4.95 - 31.05)	
**Handle**	14		1	7.14% (0.18 - 33.86)		4	28.57% (8.38 - 58.10)	
**Equipment**	30	TC2	0	0.00% (0.00)		0	0.00% (0.00)	
**Handle**	20		0	0.00% (0.00)		0	0.00% (0.00)	
**Non-Clinical**								
**Equipment**	21	Bank 1	0	0.00% (0.00)	0.706	0	0.00% (0.00)	0.926
**Handle**	9		0	0.00% (0.00)		0	0.00% (0.00)	
**Surface**	4		0	0.00% (0.00)		0	0.00% (0.00)	
**Equipment**	15	School 1	0	0.00% (0.00)		1	6.67% (0.16 - 31.94)	
**Handle**	31		0	0.00% (0.00)		1	3.23% (0.08 - 16.70)	
**Surface**	2		0	0.00% (0.00)		0	0.00% (0.00)	
**Equipment**	26	Bank 2	0	0.00% (0.00)		0	0.00% (0.00)	
**Handle**	4		0	0.00% (0.00)		0	0.00% (0.00)	
**Equipment**	20	School 2	1	5.00% (0.12 - 24.87)		1	5.00% (0.12 - 24.87)	
**Handle**	44		1	2.27% (0.05 - 12.02)		1	2.27% (0.05 - 12.02)	
**Surface Material**								
**Clinical**								
**Epoxy Resin**	26	TC1	1	3.85% (0.09 - 19.63)	0.913	1	3.85% (0.09 - 19.63)	0.881
**Fabric**	4		0	0.00% (0.00)		0	0.00% (0.00)	
**Formica**	8		0	0.00% (0.00)		1	12.50% (0.31 - 52.65)	
**Glass**	20		0	0.00% (0.00)		0	0.00% (0.00)	
**Leather**	13		0	0.00% (0.00)		0	0.00% (0.00)	
**Metal**	108		2	1.85% (0.22 - 6.52)		4	3.70% (1.01 - 9.21)	
**Plastic**	120		6	5.00% (1.85 - 10.56)		5	4.17% (1.36 - 9.45)	
**Tile**	2		0	0.00% (0.00)		0	0.00% (0.00)	
**Wood**	13		0	0.00% (0.00)		1	7.69% (0.19 - 36.02)	
**Epoxy Resin**	8	RC	0	0.00% (0.00)		1	12.50% (0.31 - 52.65)	
**Leather**	2		0	0.00% (0.00)		1	50.00% (1.25 - 98.74)	
**Metal**	30		1	3.33% (0.08 - 17.21)		6	20.00% (7.71 - 38.56)	
**Plastic**	8		0	0.00% (0.00)		1	12.50% (0.31 - 52.65)	
**Metal**	24	TC2	0	0.00% (0.00)		0	0.00% (0.00)	
**Plastic**	26		0	0.00% (0.00)		0	0.00% (0.00)	
**Non-Clinical**								
**Formica**	8	Bank 1	0	0.00% (0.00)	0.706	0	0.00% (0.00)	0.935
**Glass**	4		0	0.00% (0.00)		0	0.00% (0.00)	
**Leather**	4		0	0.00% (0.00)		0	0.00% (0.00)	
**Metal**	10		0	0.00% (0.00)		0	0.00% (0.00)	
**Plastic**	8		0	0.00% (0.00)		0	0.00% (0.00)	
**Epoxy Resin**	8	School 1	0	0.00% (0.00)		0	0.00% (0.00)	
**Glass**	2		0	0.00% (0.00)		0	0.00% (0.00)	
**Metal**	30		0	0.00% (0.00)		1	3.33% (0.08 - 17.21)	
**Plastic**	8		0	0.00% (0.00)		0	0.00% (0.00)	
**Formica**	14	Bank 2	0	0.00% (0.00)		1	7.14% (0.18 - 33.86)	
**Metal**	6		0	0.00% (0.00)		0	0.00% (0.00)	
**Plastic**	10		0	0.00% (0.00)		0	0.00% (0.00)	
**Metal**	3	School 2	0	0.00% (0.00)		0	0.00% (0.00)	
**Plastic**	41		1	2.44% (0.06 -12.85)		1	2.44% (0.06 - 12.85)	
**Wood**	20		1	5.00% (0.12 -24.87)		1	5.00% (0.12 - 24.87)	

Observations from general areas swabbed showed that, for clinical settings, SARS-CoV-2 was detected in the administration area (5.56%, 1/18), isolation wards (6.89%, 7/102), and outpatient departments (2.50%, 1/40) of TC1 in the morning. By afternoon (7 hours later), only 4.90% (5/102) of areas in the isolation still showed positive for SARS-CoV-2 RNA. At TC2, however, SARS-CoV-2 was not detected on any of the surfaces swabbed in the morning or afternoon. We detected SARS-CoV-2 RNA on 2.08% (1/48) of surfaces swabbed from the Research Centre, and a substantial increase in positivity from 2.08% (1/48) in the morning to 18.75% (9/48) in the afternoon (p-value < 0.001) [Table 1], representing a significant difference.

In non-clinical settings, we detected SARS-CoV-2 on 3.13% (2/64) of surfaces swabbed in the classroom of our second selected site, compared to none in the designated banks. In the afternoon, this persisted, and we further detected SARS-CoV-2 in the washroom of our first selected school at a rate of 25.00% (1/4) and in the banking hall of our second selected bank at a rate of 3.85% (1/26) (Table 1).

### Distribution of SARS-CoV-2 according to surfaces swabbed and materials they are made of

**Figure 2:**
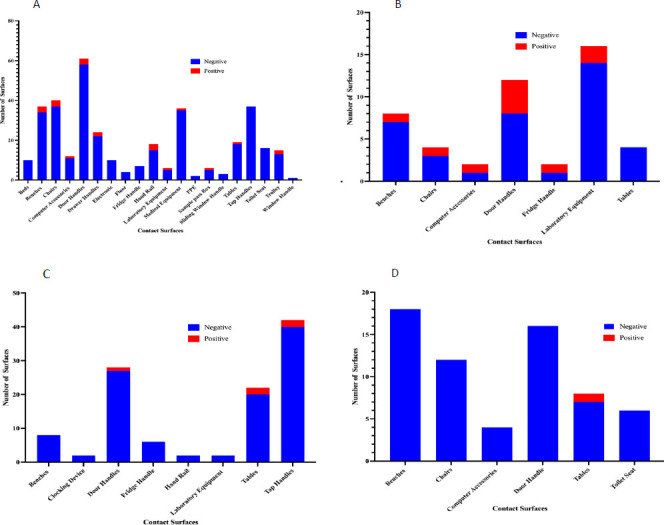
Distribution of the number of contact surfaces per site. **A** shows frequently contact surfaces in treatment centres; **B.** Research laboratory **C.** selected school; **D.** Selected bank. Results show that door handles are the most frequently contacted surfaces across both clinical and non-clinical settings

A total of 21/37 (56.7%) surfaces tested positive in the treatment centers, and 10/37 (27.0%) tested positive in the research Centre. In the treatment center this included door handles 8.11% (3/37), benches 8.11% (3/37), chairs 8.11% (3/37), handrails 8.11% (3/37), lab equipment 2.70% (1/37), computer accessories 2.70% (1/37), drawer handle 5.41% (2/37), trolley 5.41% (2/37), fridge handles 2.70% (1/37), medical equipment 2.70% (1/37), sample pass box 2.70% (1/37) and tables 2.70% (1/37) [Figure 2]. Surfaces that tested positive in non-clinical settings included: tables (50.00%, 3/6), tap handles (33.33%, 2/6), and door handles (16.67%, 1/6). Banks recorded the fewest number of SARS-CoV-2 positives on surfaces, with 1 (2.7%) table testing positive in the afternoon (Figure 2).

### Viability of SARS-CoV-2 from positive surface samples

All 37 positive surface samples were inoculated onto confluent Vero E6 cells. None of the inoculated samples showed any cell cytopathic effect after three passages. For confirmation, supernatants collected after the incubation period were tested by RT-qPCR. However, none turned out positive.

## Discussion

While numerous studies have confirmed that respiratory droplets released by infected individuals during coughing or sneezing can contaminate surfaces, the scientific community remains divided on the significance of indirect SARS-CoV-2 transmission through environmental surfaces. This observation stems from the virus's long-lasting persistence on various surfaces and its dependence on factors such as temperature and relative humidity for viability.^9^ Some studies have observed that fomites can harbour viable SARS-CoV-2 virus for periods ranging from hours to days, depending on the type of surface, with a maximum of 24 hours on cardboard surfaces and 72 hours on plastic and stainless-steel surfaces.^5^–^7^,^13^ Although SARS-CoV-2 can persist on inanimate surfaces for prolonged periods, surface disinfection procedures can efficiently inactivate the virus, and transmission can be prevented through personal preventive measures, such as washing hands and wearing face masks.^8^,^14^ Compliance, however, with these measures has become a bottleneck in the efforts to mitigate the spread of the virus. Nonetheless, Ashinyo et al (2021) reported higher compliance levels among healthcare workers than non-healthcare workers. Although non-clinical settings have received less attention from studies investigating environmental surface contamination, the risk of fomite-mediated transmission in non-healthcare settings cannot be overstated, as residents are less protected and surfaces are not disinfected as frequently as in hospital settings.^11^

To put environmental surface contamination into perspective, this study investigated the presence and infectivity of SARS-CoV-2 on frequently touched surfaces in both clinical and non-clinical settings within Accra. Clinical sites, such as hospitals or treatment centres, typically have a higher influx of patients who may be infected with SARS-CoV-2. This increased patient traffic may contribute to higher viral loads in the environment, which in turn may account for the high positivity rates. In contrast, non-clinical sites tend to have lower foot traffic of infected individuals and exposure to those who are infected, resulting in lower positivity rates. We commenced the study by observing which surfaces were frequently touched at COVID-19 treatment centres, a research laboratory representing clinical settings, and schools and banks to denote non-clinical settings. Across all these areas, we observed that door handles were the most frequently touched surfaces. In addition to door handles, tables and desks, as well as knobs on handwash stations and computer accessories, were more regularly touched. These are all surfaces that are highly utilized for work across clinical and non-clinical settings. We ultimately gathered a total of 588 swab samples from frequently touched surfaces for laboratory analysis, of which 6.3% tested positive for SARS-CoV-2.

It was also observed that surface contamination in clinical settings was higher (83% (31/37) compared with non-clinical settings, with the highest detection rates found in the isolation and treatment centres. This observation could be due to the larger sample size obtained from the clinical settings (COVID-19 Isolation and Treatment Centres and a research laboratory). Moreover, a higher level of contamination was expected within the treatment centers and research laboratories due to the presence of COVID-19 patients at these facilities. Our findings align with those of a review study, which found that the majority of surface samples (64.1%) and SARS-CoV-2 positives (17%) originated from hospitals, including a COVID-19 isolation ward and diagnostic laboratories.^10^ Research findings from Kampf et al., (2020) showed that surface disinfection procedures and strategies, such as washing hands, inactivate the SARS-CoV-2 virus and wearing face masks could subsequently limit the spread. In healthcare settings, compliance with these measures is believed to be more firmly adhered to; therefore, the widespread transmission of pathogens from surfaces could be limited.^8^ However, the consequences of surface contamination in these settings cannot be completely ignored, as it could lead to subsequent transmission.^16^

Dargahi et al., (2021) investigated SARS-CoV-2 viral viability on environmental surfaces and found cupboards, light switches, and door handles to be contaminated. Our results also suggest that door handles were the most contaminated surface with SARS-CoV-2 RNA, across all study sites sampled. Other frequently touched surfaces that were found to be highly contaminated within the clinical settings were lab equipment, fridge handles, computer accessories, benches, chairs, drawer handles, handrails, trolleys and tables. Contamination of frequently touched surfaces, including high-touch surfaces in rooms (e.g., bedrails, over-bed tables, and call-buttons) and reusable patient care equipment that is moved between rooms, can lead to: (1) transmission to the next patient who occupies the room or uses the same equipment, or (2) contamination of the hands or clothing of healthcare personnel with transmission to other patients. Again, tables and tap handles were found to be contaminated with SARS-CoV-2 RNA, in addition to door handles, within schools and banks. The high contamination of door handles could be attributed to the fact that in Ghana, most doors, though frequently touched, remain manually operated. This could account for the high level of contamination. Contamination of such surfaces has been linked to the spread of infections. It is imperative, therefore, that such surfaces be frequently decontaminated to minimise transmission. Again, as much as possible, people should avoid touching doorknobs as they have been implicated in the spread of several infectious agents.

Studies by van Doremalen et al., (2020) showed that SARS-CoV-2 was more stable on plastic and stainless steel than copper and cardboard, and that viable virus was detected up to 72 hours after application to these surfaces. Results from this study also found higher positivity on plastic and metal surfaces compared to wood and other materials. Further confirming that viruses exhibit greater persistence on non-porous materials compared to porous surfaces. A large majority of door handles are made of stainless steel. Therefore, it is entirely possible that infectious viruses survived longer on such materials, due to their non-porous nature and delayed delivery evaporation rate, as demonstrated by many studies.^13^,^18^,^19^

We found that, in both the clinical and non-clinical sectors, more samples tested positive in the afternoon (4.08%, 12/294 versus 8.50%, 25/294, respectively), although the difference was statistically significant (p-value = 0.027). Lower positivity in the morning compared to the afternoon may be explained by the fact that appropriate cleaning and decontamination are completed before the workday begins. Although a study found the SARS-CoV-2 virus to be inactivated at high temperatures^20^ This study still detected virus RNA on some surfaces in the afternoon, when temperatures are known to be high at 27.5 °C and relative humidity is 69.00%. This high positivity recorded in the afternoon may be a result of viral deposition by personnel during working hours. Aerosolgenerating activities and procedures during working hours could also contribute to surface contamination with SARS-CoV-2 in the afternoon. We could also attribute this finding to reduced disinfection procedures in the afternoon, mainly because most people clean before and after all work has been done. Accordingly, a study showed that samples collected before disinfection yielded more positives compared to those taken after disinfection.^17^ This observation, in conjunction with our study findings, underscores the importance of maintaining regular surface decontamination practices.

Viable SARS-CoV-2 was not recovered from positive samples in this study. This finding is consistent with a few studies that have been unable to recover viable SARS-CoV-2 from swabbed surfaces using cell culture.^11^,^21^,^22^ The difficulty in recovering viable and infectious SARS-CoV-2 from swabbed surfaces may be due to low viral concentrations (corresponding to high CT values) and harsh environmental factors that can inactivate and destabilise the virus outside the living host. Detection of viral RNA does not indicate viability or assurance of recovery in culture, nor can it be relied upon solely to determine viral transmission. Contradicting the findings of Marcenac et al., CPE was observed on cells from a recovered SARS-CoV-2 sample collected from a nightstand. This may be due to a high viral load (indicated by low cycle threshold) for that sample (26.4), compared to a low viral load (high cycle threshold values) recorded in our study. The same study found no CPE in positive samples with Ct values above 30, further supporting our findings.

## Conclusion

This study demonstrates the presence of SARS-CoV-2 on frequently touched surfaces in both clinical and non-clinical settings, including schools and banks. Although the study was unable to recover live virus from positive samples, the importance of these findings cannot be overstated. The presence of SARS-CoV-2 on frequently touched surfaces, such as doorknobs and tables in schools, may have contributed to the transmission of the virus in Ghana during the pandemic. Further studies are, however, needed to determine the persistence of SARS-CoV-2 on frequently touched surfaces in such a tropical environment.

## Figures and Tables

**Table 2:** Overall positivity of SARS-CoV-2 on surface material

Characteristics	Contact Surfaces	Surface Material	N	Morning		Afternoon	
Study Sites				n	%	n	%
**Treatment centers (TC1 and TC2)**	Beds	Leather	10	0	0%	0	0%
	Benches	Epoxy Resin	26	1	4%	1	4%
		Formica	8	0	0%	1	13%
		Plastic	1	0	0%	0	0%
		Wood	2	0	0%	0	0%
	Chairs	Leather	1	0	0%	0	0%
		Plastic	39	1	3%	2	5%
	Computer Accessory	Plastic	12	0	0%	1	8%
	Door Handle	Metal	61	1	2%	2	3%
	Drawer Handle	Metal	17	1	6%	0	0%
		Wood	7	0	0%	1	14%
	Electronic	Plastic	10	0	0%	0	0%
	Floor	Leather	2	0	0%	0	0%
		Tile	2	0	0%	0	0%
	Fridge Handle	Metal	1	0	0%	0	0%
		Plastic	6	0	0%	0	0%
	Hand Rails	Plastic	18	3	17%	0	0%
	Lab Equipment	Metal	6	0	0%	1	17%
	Medical Equipment	Fabric	4	0	0%	0	0%
		Glass	20	0	0%	0	0%
		Metal	6	0	0%	0	0%
		Plastic	6	1	17%	0	0%
	PPE	Plastic	2	0	0%	0	0%
	Sample Pass Box	Metal	6	0	0%	1	17%
	Sliding Window Handle	Plastic	3	0	0%	0	0%
	Tables	Plastic	16	1	6%	0	0%
		Wood	3	0	0%	0	0%
	Tap Handle	Metal	35	0	0%	0	0%
		Plastic	2	0	0%	0	0%
	Toilet Seat	Plastic	16	0	0%	0	0%
	Trolley	Plastic	14	0	0%	2	14%
		Wood	1	0	0%	0	0%
	Window Handle	Plastic	1	0	0%	0	0%
**RESEARCH Center**	Benches	Epoxy Resin	8	0	0%	1	13%
	Chairs	Leather	2	0	0%	1	50%
		Plastic	2	0	0%	0	0%
	Computer Accessory	Plastic	2	0	0%	1	50%
	Door Handle	Metal	12	1	8%	3	25%
	Fridge Handle	Metal	2	0	0%	1	50%
	Lab Equipment	Metal	16	0	0%	2	13%
	Tables	Plastic	4	0	0%	0	0%
**SCHOOLS**	Benches	Epoxy Resin	8	0	0%	0	0%
	Clocking Device	Glass	2	0	0%	0	0%
	Door Handle	Metal	27	0	0%	1	4%
		Plastic	1	0	0%	0	0%
	Fridge Handle	Plastic	6	0	0%	0	0%
	Hand Rails	Metal	2	0	0%	0	0%
	Lab Equipment	Metal	2	0	0%	0	0%
	Tables	Plastic	2	0	0%	0	0%
		Wood	20	1	5%	1	5%
	Tap Handle	Metal	2	0	0%	0	0%
		Plastic	40	1	3%	1	3%
**BANK**	Benches	Formica	18	0	0%	0	0%
	Chairs	Leather	4	0	0%	0	0%
		Plastic	8	0	0%	0	0%
	Computer Accessory	Plastic	4	0	0%	0	0%
	Door Handle	Metal	16	0	0%	0	0%
	Tables	Formica	4	0	0%	0	25%
		Glass	4	0	0%	0	0%
	Toilet Seat	Plastic	6	0	0%	0	0%

## References

[1] Cui J, Li F, Shi ZL (2019). Origin and evolution of pathogenic coronaviruses. Nat Rev Microbiol.

[2] WHO (2023). World Health Organisation COVID-19 Dashboard.

[3] GHS (2023). Ghana Health Service COVID-19 dashboard.

[4] Organisation WH (2020). WHO declares COVID-19 outbreak a pandemic.

[5] Chia PY, Coleman KK, Tan YK (2020). Detection of air and surface contamination by SARS-CoV-2 in hospital rooms of infected patients. Nat Commun.

[6] Zheng J (2020). SARS-CoV-2: an Emerging Coronavirus that Causes a Global Threat. Int J Biol Sci.

[7] Zou L, Ruan F, Huang M (2020). SARS-CoV-2 Viral Load in Upper Respiratory Specimens of Infected Patients. N Engl J Med.

[8] Ashinyo ME, Dubik SD, Duti V (2021). Infection prevention and control compliance among exposed healthcare workers in COVID-19 treatment centers in Ghana: A descriptive cross-sectional study. Tu WJ, ed. PLoS One.

[9] Moore G, Rickard H, Stevenson D (2021). Detection of SARS-CoV-2 within the healthcare environment: a multi-centre study conducted during the first wave of the COVID-19 outbreak in England. J Hosp Infect.

[10] Gonçalves J, da Silva PG, Reis L (2021). Surface contamination with SARS-CoV-2: A systematic review. Sci Total Environ.

[11] Ong SWX, Lee PH, Tan YK (2021). Environmental contamination in a coronavirus disease 2019 (COVID-19) intensive care unit—What is the risk?. Infect Control Hosp Epidemiol.

[12] Ghana Statistical Service (GSS) (2021). 2021 Population and Housing Census. Ghana Stat Serv.

[13] van Doremalen N, Bushmaker T, Morris DH (2020). Aerosol and Surface Stability of SARS-CoV-2 as Compared with SARS-CoV-1. N Engl J Med.

[14] Seif Faezeh, Noorimotlagh Z, Mirzaee SA (2021). The SARS-CoV-2 (COVID-19) pandemic in hospitals: An insight into environmental surface contamination, disinfectants' efficiency, and estimation of plastic waste production. Environ Res.

[15] Kampf G, Todt D, Pfaender S, Steinmann E (2020). Persistence of coronaviruses on inanimate surfaces and their inactivation with biocidal agents. J Hosp Infect.

[16] Braunstein GD, Schwartz L, Hymel P, Fielding J (2021). False Positive Results With SARS-CoV-2 RT-PCR Tests and How to Evaluate a RT-PCR-Positive Test for the Possibility of a False Positive Result. J Occup Environ Med.

[17] Dargahi A, Jeddi F, Vosoughi M (2021). Investigation of SARS CoV-2 virus in environmental surface. Environ Res.

[18] Aboubakr HA, Sharafeldin TA, Goyal SM (2021). Stability of SARS-CoV-2 and other coronaviruses in the environment and on common touch surfaces and the influence of climatic conditions: A review. Transbound Emerg Dis.

[19] Watanabe M, Ohnishi T, Arai S (2022). Survival of SARS-CoV-2 and bovine coronavirus on common surfaces of living environments. Sci Rep.

[20] Riddell S, Goldie S, Hill A, Eagles D, Drew TW (2020). The effect of temperature on persistence of SARS-CoV-2 on common surfaces. Virol J.

[21] Ben-Shmuel A, Brosh-Nissimov T, Glinert I (2020). Detection and infectivity potential of severe acute respiratory syndrome coronavirus 2 (SARS-CoV-2) environmental contamination in isolation units and quarantine facilities. Clin Microbiol Infect.

[22] Döhla M, Schulte B, Wilbring G (2022). SARS-CoV-2 in Environmental Samples of Quarantined Households. Viruses.

[23] Marcenac P, Park GW, Duca LM (2021). Detection of SARS-CoV-2 on Surfaces in Households of Persons with COVID-19. Int J Environ Res Public Health.

